# Prevalence and factors associated with underutilization of antenatal care services in Nigeria: A comparative study of rural and urban residences based on the 2013 Nigeria demographic and health survey

**DOI:** 10.1371/journal.pone.0197324

**Published:** 2018-05-21

**Authors:** Emmanuel Olorunleke Adewuyi, Asa Auta, Vishnu Khanal, Olasunkanmi David Bamidele, Cynthia Pomaa Akuoko, Kazeem Adefemi, Samson Joseph Tapshak, Yun Zhao

**Affiliations:** 1 Statistical and Genomic Epidemiology Laboratory, Institute of Health and Biomedical Innovation, Queensland University of Technology, Brisbane, Australia; 2 School of Pharmacy and Biomedical Sciences, University of Central Lancashire, Preston, United Kingdom; 3 Nepal Development Society, Butwal, Nepal; 4 Drug Research and Production Unit, Faculty of Pharmacy, Obafemi Awolowo University, Ile-Ife, Osun State, Nigeria; 5 School of Nursing, Faculty of Health, Queensland University of Technology, Brisbane, Australia; 6 Health and Social Relief Initiative, Ilorin, Kwara State, Nigeria; 7 Department of Obstetrics and Gynaecology, Chivar Specialist Hospital and Urology Centre LTD, Abuja, Nigeria; 8 Department of Epidemiology and Biostatistics, School of Public Health, Curtin University, Bentley Campus, Perth, Australia; Seoul National University College of Medicine, REPUBLIC OF KOREA

## Abstract

**Introduction:**

Antenatal care (ANC) is a major public health intervention aimed at ensuring safe pregnancy outcomes. In Nigeria, the recommended minimum of four times ANC attendance is underutilized. This study investigates the prevalence and factors associated with underutilization of ANC services with a focus on the differences between rural and urban residences in Nigeria.

**Methods:**

We analyzed the 2013 Nigeria Demographic and Health Survey dataset with adjustment for the sampling weight and the cluster design of the survey. The prevalence of underutilization of ANC was assessed using frequency tabulation while associated factors were examined using Chi-Square test and multivariable logistic regression analysis.

**Results:**

The prevalence of underutilization of ANC was 46.5% in Nigeria, 61.1% in rural residence and 22.4% in urban residence. The North-West region had the highest prevalence of ANC underuse in Nigeria at 69.3%, 76.6% and 44.8% for the overall, rural and urban residences respectively. Factors associated with greater odds of ANC underuse in rural residence were maternal non-working status, birth interval < 24 months, single birth type, not listening to radio at all, lack of companionship to health facility and not getting money for health services. In urban residence, mothers professing Islam, those who did not read newspaper at all, and those who lacked health insurance, had greater odds of ANC underuse. In both rural and urban residence, maternal and husband’s education level, region of residence, wealth index, maternal age, frequency of watching television, distance to- and permission to visit health facility were significantly associated with ANC underuse.

**Conclusions:**

Rural-urban differences exist in the use of ANC services, and to varying degrees, factors associated with underuse of ANC in Nigeria. Interventions aimed at addressing factors identified in this study may help to improve the utilization of ANC services both in rural and urban Nigeria. Such interventions need to focus more on reducing socioeconomic, geographic and regional disparities in access to ANC in Nigeria.

## Introduction

Promoting optimum health for women and reducing maternal and childhood mortalities have remained a significant interest for the international community for decades [1, 2]. This special interest was demonstrated by the high priority accorded maternal and child health care in the Millennium Development Goals (MDGs), and more recently, the Sustainable Development Goals (SDGs) [3]. Between 1990 and 2015, appreciable gains were recorded in the global maternal-child-health care with 43.9% and 48% reduction in Maternal Mortality Ratio (MMR) and under-five mortality rate (U5MR), respectively–thanks to the MDGs initiative [1, 2]. Despite these impressive achievements, the challenge of maternal, neonatal and other childhood mortalities remain considerably high in several developing countries with wide-ranging disparities between and within different population groups [2, 3].

Nigeria shares a disproportionately high burden of the global maternal and neonatal mortalities, ranking as the first and the second country in the world for the highest number of deaths among mothers and neonates, respectively [2, 4, 5]. These poor indices may be linked with the low utilization of maternal healthcare services in the country [4, 6]. Recent findings demonstrating a correlation between maternal healthcare services utilization, MMR, and neonatal mortality rates support this premise [7, 8]. Specifically, studies demonstrate that ANC attendance may protect against neonatal mortality and countries with low ANC attendance have higher MMR [8–10]. Going by this consistent and growing body of evidence, ANC, one of the pillars of the ‘safe motherhood initiative’[11], remains a major public health intervention for preventing maternal and neonatal mortality, worldwide.

ANC, also known as ‘health care during pregnancy’, entails periodic visits by pregnant women to designated health centers staffed and equipped for maternity services [12, 13]. The World Health Organization (WHO) promotes a ‘focussed’ ANC package which requires a minimum of four antenatal attendance affording pregnant women the opportunity of appropriate counselling, micronutrient supplementation (folic acid and iron), medical screening, vaccination and preventive treatment for malaria, all aimed at ensuring safe pregnancy outcomes [12–14]. Conditions such as hepatitis, human immunodeficiency virus, high blood pressure and gestational diabetes are usually screened for during ANC visit [12, 14, 15].

Furthermore, ANC attendance assists in the early detection of high-risk pregnancies as women with risk factors suggestive of possible obstetric complication(s) are identified through careful history taking and appropriate medical screening for specialized and individualized management plan(s) [12, 14]. Increased chances of institutional delivery and hence prevention and/or treatment of the leading causes of maternal and early neonatal mortality through timely access to-/utilization of emergency obstetric care services are parts of the benefits of ANC attendance [6, 14]. A recent study in Nigeria indicates that women who attended ANC had nine to ten times increased odds of utilizing healthcare facility for childbirth in rural and urban residences [6]. And, the greater the ANC attendance, the less likely was home delivery [6]. Hence, the imperatives of promoting ANC use in the country.

Its numerous benefits notwithstanding, ANC attendance of the recommended minimum of four times (focused ANC) [13] remains low in Nigeria [4, 16]. The report of the 2013 Nigeria Demographic and Health Survey (NDHS) indicates that in the five years preceding the survey, only 51.1% of women had four or more ANC attendance in the country [4]. This ANC prevalence is far below the recommended target of 90% attendance [13] and comparatively lower than the case in similar developing countries like Cameroon (62.9%) [17], Ghana (87%) [18], and Peru (94.4%) [17]. Given the importance of ANC–dual roles of protecting against maternal and neonatal mortality–evidence-based knowledge on factors associated with its low utilization, particularly, with regards to the within-population differences is critical to the speedy realization of SDG 3 in Nigeria. Unfortunately, studies with a major focus on this crucial subject are limited in the country.

So far, a few nationally representative studies have assessed factors associated with the utilization of ANC in Nigeria. For example, a previous analysis of the pooled 2013 NDHS [16] reported a significant association between ANC visits and maternal age, maternal working status, maternal education level, husband’s education level, wealth index, rural-urban residence, region of residence and religion. Similarly, a study investigating the barriers to ANC uptake in Nigeria reported ‘getting money to go’, ‘distance from health facility’, ‘availability of transport to the facilities’ as the three leading barriers [19]. However, all these and similar studies are limited in that they focussed primarily on the national estimates, using pooled datasets which may mask the within-population variations such as the rural-urban differences.

Based on the analysis of the 2008 NDHS, a study has assessed the determinants of “urban-rural differentials” in ANC use in Nigeria [20]. However, the title of the study could be misleading as rural-urban stratification was not used in the multivariable analysis [20]. Indeed, the author only adjusted for ‘rural-urban residence’ as an independent variable, hence, the determinants of ANC reported applied only to the overall Nigerian population in the five years preceding the 2008 NDHS. Thus, factors associated with ANC use/underuse in rural and urban residences in Nigeria remain unclear.

Given the notable sociocultural and socioeconomic disparities between rural and urban communities in Nigeria [4], the present study aims to assess the rural-urban differences in the prevalence and factors associated with ANC underuse in the country using the most recent NDHS, 2013. This study objective agrees with the recent WHO’s framework for monitoring progress towards universal health coverage as well as the recommendation of using disaggregated studies as an evidence-based approach to bridging equity gaps across geographic divides [2, 21, 22]. A conceptual framework adapted from Andersen’s behavioral model [23] guided the study with factors tailored for the Nigerian context. Findings are expected to provide further evidence which may inform targeted interventions aimed at improving ANC attendance and subsequently contribute towards achieving SDG 3 in Nigeria.

## Materials and methods

This study was based on a secondary analysis of the 2013 NDHS, a cross-sectional and nationally representative data that are freely available online (http://www.dhsprogram.org) with permission from ICF international, USA [4]. The Nigerian National Health Research Ethics Committee granted ethical approval for the conduct of the survey [4]. Participants provided informed written consent, or had it provided on their behalf by parents/guardians if they were younger than 18 years at the time of the survey [4]. The data utilized in the present study were fully anonymized before we accessed them with no information identifying survey participants. No additional ethical clearance was required for the conduct of the present study. Permission to use the data was sought and obtained from ICF International, USA.

Prior to the 2013 –the latest and most current survey in the series–there have been four previous editions of NDHS beginning from 1990 [4]. The 2013 NDHS was implemented by the Nigerian Population Commission with support from many development partners including the United Kingdom Department for International Development, the United States Agency for International Development, and the United Nations Populations Fund [4]. The survey aimed at providing current and up-to-date data on marriage, awareness, and use of family planning methods, maternal and child vaccination coverage, nutritional status of women and children, healthcare services utilization, maternal and childhood mortalities, and so on, in Nigeria [4]. Technical support for the survey was provided by ICF International, USA, through the MEASURE Demographic and Health Survey program [4].

Sample collection in the 2013 NDHS was carried out through a stratified three-stage cluster sampling, the design of which consisted of 904 clusters– 372 and 532 in urban and rural areas, respectively [4]. A representative sample of 40680 households (16740 in urban areas and 23940 in rural areas) was selected for the survey [4]. Interviewer-administered structured questionnaires were utilized for data collection and three types namely women’s, men’s and households’ questionnaires were used [4]. Eligible to be interviewed in the survey were women aged 15 to 49 years old present in each of the selected households for at least a night prior to the survey, as well as men of the same age category but present in the alternate households for at least a night before the survey [4].

Out of the 38904 households occupied as at fieldwork time of the survey (16070 in urban areas and 22834 in rural areas), only 38 522 were successfully interviewed (15 859 in urban areas and 22 663 in rural areas), yielding a response rate of 99% (98.7% in urban areas and 99.3% in rural areas) [4]. A comprehensive report on the sampling procedures, settings, questionnaires and the design of the 2013 NDHS has previously been published [4]. The data analyzed in the present study were restricted to those of 19652 mothers with complete information on ANC attendance/non-attendance for their most recent childbirth in the five years preceding the 2013 survey. Where appropriate, the terms underutilization and underuse were used interchangeably.

### Study factors

The main outcome variable for this study was underutilization of ANC service defined as antenatal attendance of less than the recommended minimum of four times [4, 24, 25]. This definition follows the WHO recommendation of a focused ANC model which recommends at least four ANC attendance for pregnant women with no complication, to better optimize the benefits of ANC services. Women with associated ill-health or those with/at risk of complication(s) are required to make a greater number of attendance [12–14]. Antenatal services as used in the present study are pregnancy-related care provided by skilled healthcare professionals including doctors, nurses, midwives, and auxiliary nurses/midwives [4].

Underutilization of ANC as our outcome of interest encompasses non-attendance of ANC as well as attendance of less than the recommended minimum of four times [4, 26, 27]. The ANC attendance variable captured in the 2013 NDHS (for the most recent live births within five years leading to the survey) was dichotomized as less than four times attendance (< 4, underutilization, coded as “1”) and at least four times ANC attendance (≥ 4, utilization, coded as “0”) for use in binary logistic regression analyses. Mothers who responded to the question “how many times did you receive antenatal care during this pregnancy” by saying ‘don’t know’ as well as those with missing information were excluded in analyses.

**Fig 1:**
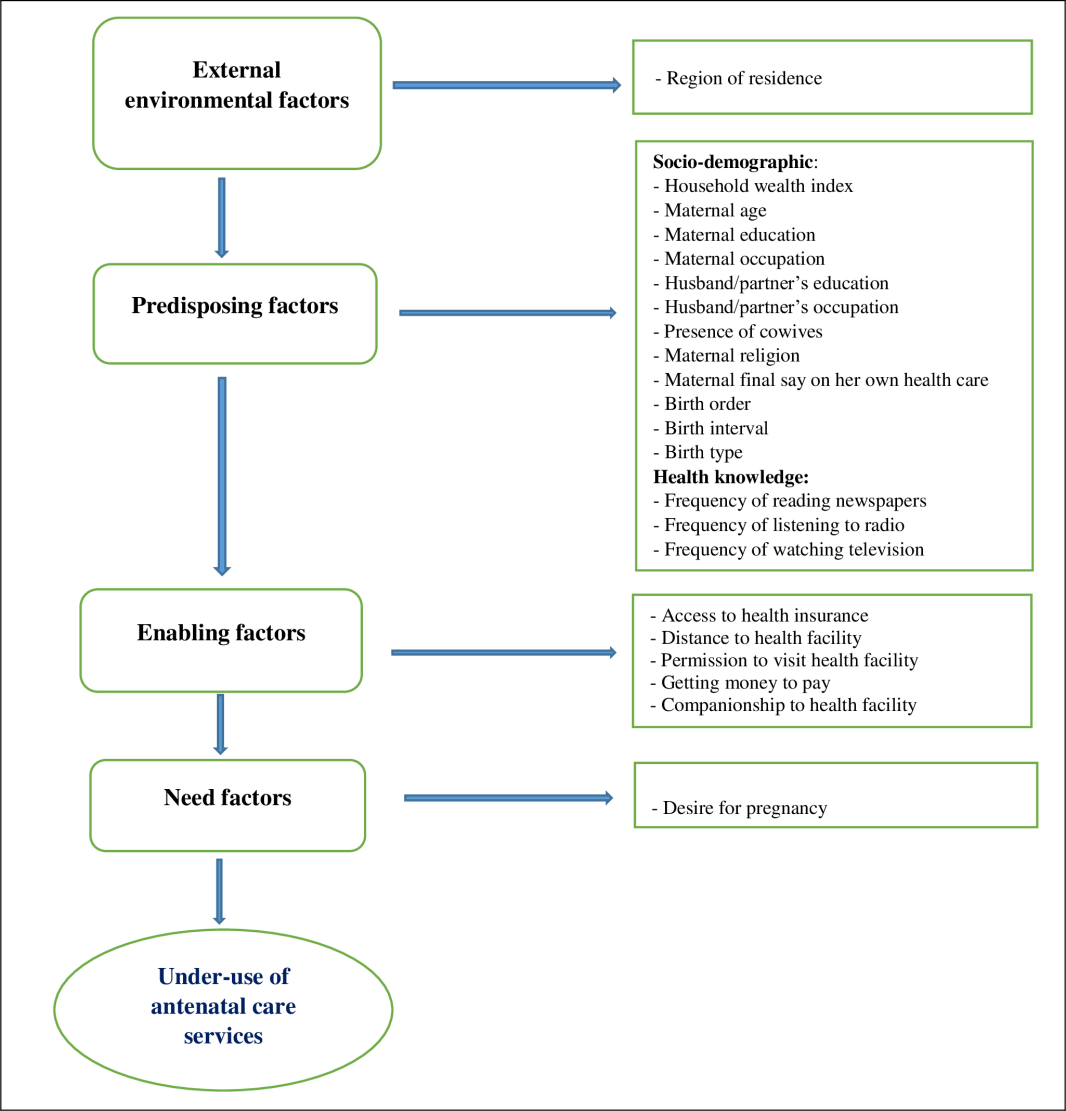
Theoretical framework for studying factors associated with underuse of antenatal care services in Nigeria (adapted from Andersen’s behavioral model [23]).

In line with previous studies [26, 28], we adapted the Andersen’s behavioral model [23] as a conceptual framework in selecting explanatory variables for this study (Fig 1) with consideration of the available information in the 2013 NDHS [4]. In all, twenty-three explanatory variables, broadly classified into four: external environmental factors, predisposing factors, enabling factors and need factors, were assessed (Fig 1). The external environmental factors were residence (rural and urban) as well as the region of residence (categorized according to the six geopolitical zones in Nigeria: North-Central, North-East, North-West, South-East, South-South, and South-West).

The predisposing factors were sub-classed into two namely, socio-demographic and health knowledge factors. The socio-demographic factors assessed included household wealth index, a composite indicator of socioeconomic status, derived from the principal component analysis of households’ assets. The variable was re-categorized from the five levels in the 2013 NDHS to three as follows: poor = “poorest” + “poorer,” middle = “middle,” rich = “richer” + “richest”[4, 6]. Other socio-demographic factors were maternal age (< 20, 20–34, ≥ 35 years) [5], maternal and husband/partner’s education level (none, primary and secondary/higher) [27], maternal and husband/partner’s working status (working, not working) [6], presence of co-wives (no co-wife, other co-wives) [16] and maternal religion (Christianity, Islam, Traditional/other) [6, 16]. Mother’s final say on her own health (respondent alone, respondent and husband/partner, husband/partner/someone else/other) [27], birth order (1, 2–3, ≥ 4) [3], preceding birth interval (< 24, ≥ 24 months) [3] and birth type (singleton, multiple) were equally classified under socio-demographic predisposing factors.

Three variables which are related to the level of media exposure–frequency of reading newspapers/magazines, listening to radio and watching television (all categorized as: not at all, less than once a week, and at least once a week) [26, 29] were classified under health knowledge factors. Enabling factors, on the other hand, comprised of variables which may encourage health care services utilization. These included access to health insurance (yes, no) [16], distance to health facility, companionship to a health facility, getting money to pay for health services, and getting permission to visit health facility (all categorized as: a big problem, not a big problem) [26, 27]. Lastly, the ‘desire for pregnancy’ (categorized as: then, later, no more) was assessed as a need factor [16, 27].

### Statistical analysis

The prevalence (in %) of underuse of ANC (< 4 times ANC attendance) alongside its 95%CI was estimated against the explanatory variables for the overall, rural and urban residences using frequency tabulation. To assess the unadjusted association between the outcome and explanatory variables, Chi-Square test was performed, and p-values reported by comparing the prevalence of underuse of ANC between variable categories. The adjusted likelihood of ANC underuse was assessed using multivariable binary logistic regression analyses accounting for the effects of all other explanatory variables included in the models.

The hierarchical modeling method [30] was used in the multivariable model building process. Variables that satisfied the inclusion criterion of p < 0.20 in the Chi-Square test were selected for inclusion in model building in line with practice in previous studies [3, 5, 26]. The first model combined ‘external environmental factor’ with the ‘predisposing factors’ (Fig 1). Backward elimination procedure was performed, and variables were retained for the next model if they were significantly associated with underuse of ANC at 5% significance level (p < 0.05). The second model comprised of variables retained in the first model together with the ‘enabling factors’; backward elimination was similarly carried out to identify factors that were significantly associated with the outcome variable at p < 0.05. To obtain the final parsimonious model, variables retained in the second model were assessed against the outcome variable with the inclusion of the ‘need factor’. Adjusted odds ratios (AOR), their 95%CI and p-values for variables retained in the final parsimonious model were reported.

The multivariable binary logistic regression analysis procedures were carried out first for the overall Nigerian population (pooled dataset) and subsequently replicated for data disaggregated by rural and urban residences. The backward elimination modeling was tested against potential confounders or factors previously reported in studies so as not to miss any statistically/clinically significant/important variable. Data management and all analyses were carried out using the Statistical Package for Social Sciences (SPSS), version 21 released 2012 (IBM, Armonk, NY, USA). The Complex Sample Analysis method was used in all analyses to account for the sampling weight and the multi-stage cluster design of the 2013 NDHS [31].

## Results

Table 1 describes the characteristics of the sample population. A total of 19652 mothers was included in this study of which 64.9% were rural and the rest were urban residents. Approximately half of the mothers in the overall population did not have formal education (48.5%), more than two thirds (68.6%) were working, nearly half (46.0%) were from poor households, and about two-thirds were aged 20–34 years old. Only 5.2% of the mothers could decide by themselves on issues related to their own health care (final say on own health). Following the rural-urban disaggregation of the overall data, the percentages differ in the two residences with urban women having better profile compared to their rural counterparts (Table 1).

**Table 1:** Sample characteristics and prevalence of ANC underutilization.

Factors	Nigeria (overall)			Rural Nigeria			Urban Nigeria		
	n (%)^a^	Prevalence of ANC underuse (<4 times ANC attendance) ^a^		n (%)^a^	Prevalence of ANC underuse (<4 times ANC attendance) ^a^		n (%)^a^	Prevalence of ANC underuse (<4 times ANC attendance) ^a^	
		% (95%CI)	P-Value		% (95%CI)	P-Value		% (95%CI)	P-Value
***External environmental factors***									
**Region of residence**			< 0.001*			< 0.001*			< 0.001*
North-Central	3025 (14.2)	43.3 (38.2–48.6)		2112 (16.9)	51.4 (45.0–57.8)		913 (9.3)	16.0 (11.4–22.0)	
North-East	3917 (16.9)	60.3 (55.7–64.7)		3102 (19.7)	66.7 (61.6–71.4)		815 (11.6)	40.2 (31.9–49.1)	
North-West	6145 (37.0)	69.3 (65.9–72.5)		4915 (44.0)	76.6 (72.9–79.9)		1230 (24.0)	44.8 (37.6–52.2)	
South-East	1653 (8.3)	13.6 (10.9–16.8)		585 (4.0)	13.3 (10.6–16.5)		1068 (16.3)	13.8 (10.2–18.4)	
South-South	2343 (9.2)	32.0 (28.9–35.3)		1649 (9.3)	39.1 (35.0–43.4)		694 (8.9)	18.2 (14.2–23.0)	
South-West	2569 (14.5)	10.2 (6.9–14.9)		786 (6.1)	22.6 (11.9–38.5)		1783 (29.9)	5.5 (4.2–7.2)	
**Rural-urban residence**			< 0.001*						
Rural	13149 (64.9)	61.1 (58.5–63.5)							
Urban	6503 (35.10	22.4 (20.0–25.1)							
***Predisposing factors***									
**Maternal education level**			< 0.001*			< 0.001*			< 0.001*
Secondary/higher	6618 (32.5)	16.8 (15.4–18.4)		2863 (19.0)	26.7 (24.1–29.3)		3755 (57.4)	10.8 (9.2–12.6)	
Primary	3992 (19.0)	37.2 (34.7–39.9)		2630 (18.1)	46.2 (42.7–49.7)		1362 (20.8)	22.8 (19.9–26.0)	
None	9042 (48.5)	72.1 (69.7–74.3)		7656 (62.9)	75.7 (73.1–78.1)		1386 (21.8)	52.7 (47.0–58.2)	
**Maternal working status**			< 0.001*			< 0.001*			< 0.001*
Not working	6074 (31.4)	58.5 (55.8–61.2)		4431 (34.5)	69.5 (66.3–72.4)		1643 (25.7)	31.3 (27.5–35.4)	
Working	13493 (68.6)	42.5 (40.3–44.7)		8656 (65.5)	56.6 (53.8–59.4)		4837 (74.3)	19.4 (17.0–22.1)	
**Husband/partner’s education level**			< 0.001*			< 0.001*			< 0.001*
Secondary/higher	8167 (41.7)	24.0 (22.3–25.7)		4040 (29.0)	36.0 (33.4–38.8)		4127 (65.4)	14.0 (12.3–15.9)	
Primary	3600 (18.4)	40.6 (37.8–43.5)		2468 (18.6)	50.8 (47.2–54.3)		1132 (18.0)	21.1 (17.7–24.9)	
None	7184 (39.8)	76.2 (73.8–78.4)		6179 (52.4)	79.3 (76.8–81.6)		1005 (16.5)	57.7 (51.5–63.6)	
**Husband/partner’s working status**						0.001*			0.283
Not working	181 (0.8)	28.6 (21.4–37.1)	< 0.001*	95 (0.6)	40.2 (28.4–53.2)		86 (1.1)	16.6 (9.2–28.2)	
Working	18833 (99.2)	48.1 (46.0–50.1)		12663 (99.4)	61.6 (59.1–64.1)		6170 (98.9)	22.6 (20.1–25.4)	
**Wealth index**			< 0.001*			< 0.001*			< 0.001*
Rich	6826 (34.9)	17.0 (15.4–18.8)		2130 (13.9)	23.4 (20.5–26.6)		4696 (73.7)	14.8 (12.8–17.0)	
Middle	3975 (19.1)	41.1 (38.3–44.0)		2861 (20.9)	42.1 (38.8–45.6)		1114 (16.0)	38.6 (33.6–43.9)	
Poor	8851 (46.0)	73.3 (70.8–75.6)		8158 (65.2)	75.1 (72.6–77.5)		693 (10.3)	51.7 (43.5–59.9)	
**Maternal age**			< 0.001*			< 0.001*			0.004*
≥ 35	5378 (26.6)	45.9 (43.4–48.4)		3514 (25.9)	59.5 (56.4–62.6)		1864 (28.4)	22.8 (19.9–25.9)	
20–34 years	13028 (66.7)	46.6 (44.5–48.8)		8600 (65.9)	60.6 (57.9–63.2)		4428 (68.2)	21.7 (19.2–24.5)	
< 20 years	1246 (6.5)	63.1 (59.1–67.0)		1035 (8.2)	69.6 (65.5–73.5)		211 (3.4)	34.0 (25.3–43.9)	
**Maternal religion**			< 0.001*			< 0.001*			< 0.001*
Christianity	8005 (37.0)	25.1 (22.9–27.4)		4599 (29.9)	38.1 (34.9–41.5)		3406 (50.2)	10.7 (9.2–12.5)	
Traditional/other	301 (1.6)	60.6 (50.1–70.2)		218 (1.9)	70.8 (67.8–73.7)		83 (1.0)	25.3 (15.7–38.3)	
Islam	11346 (61.4)	60.7 (58.1–63.2)		8332 (68.3)	71.3 (59.2–80.9)		3014 (48.8)	34.4 (30.6–38.3)	
**Birth order**			< 0.001*			< 0.001*			< 0.001*
1	3541 (18.0)	41.2 (38.5–43.9)		2234 (17.0)	56.3 (52.9–59.7)		1307 (19.9)	17.3 (14.7–20.3)	
2–3	6055 (31.3)	44.3 (41.8–46.8)		3810 (29.6)	59.9 (56.7–63.1)		2245 (34.5)	19.5 (16.4–23.0)	
≥ 4	10056 (50.7)	51.7 (49.6–53.9)		7105 (53.5)	63.2 (60.6–65.7)		2951 (45.6)	26.9 (24.0–30.0)	
**Preceding birth interval**			0.013**			< 0.001*			0.975
< 24 months	3049 (19.1)	51.3 (48.5–54.1)		2072 (19.0)	65.7 (62.5–68.8)		977 (19.2)	23.7 (20.0–27.8)	
≥ 24 months	13017 (80.9)	48.4 (46.3–50.5)		8816 (81.0)	61.2 (58.6–63.8)		4201 (80.8)	23.6 (20.9–26.6)	
**Birth type**			< 0.001*			0.001*			0.995
Multiple	383 (1.9)	39.1 (33.5–45.0)		237 (1.8)	49.7 (42.3–57.2)		146 (2.1)	22.4 (15.4–31.5)	
Single	19269 (98.1)	47.7 (45.6–49.7)		12912 (98.2)	61.3 (58.7–63.7)		6357 (97.9)	22.4 (20.0–25.1)	
**Presence of co-wives**			< 0.001*			< 0.001*			< 0.001*
No co-wife	12369 (67.5)	42.1 (40.1–44.2)		7666 (61.3)	57.4 (54.7–60.1)		4703 (79.0)	19.8 (17.4–22.5)	
Other wife/wives	6009 (32.5)	61.1 (58.3–63.8)		4699 (38.7)	69.0 (65.9–72.0)		1310 (21.0)	33.3 (28.6–38.4)	
**Final say on own health**			< 0.001*			< 0.001*			< 0.001*
Respondent alone	1021 (5.3)	22.4 (19.2–25.9)		480 (3.2)	39.0 (33.6–44.8)		541 (9.2)	11.3 (8.5–14.8)	
Respondent and husband/partner	5801 (30.7)	30.0 (27.7–32.3)		3210 (24.5)	45.5 (41.8–49.1)		2591 (42.4)	13.1 (11.1–15.5)	
Husband/partner/someone else/other	11652 (64.0)	59.1 (56.8–61.3)		8727 (72.3)	68.4 (65.8–70.9)		2925 (48.5)	32.9 (29.4–36.6)	
***Health knowledge factors***									
**Frequency of reading newspaper/magazine**			< 0.001*			< 0.001*			< 0.001*
Not at all	16681 (85.8)	53.2 (51.1–55.4)		12019 (93.2)	64.1 (61.5–66.6)		4662 (72.3)	27.4 (24.4–30.6)	
< once a week	1642 (8.3)	14.2 (12.1–16.5)		621 (4.0)	21.5 (17.9–25.6)		1021 (16.2)	10.8 (8.4–13.7)	
≥ once a week	1207 (5.9)	10.9 (8.6–13.8)		420 (2.8)	17.5 (13.3–22.6)		787 (11.5)	8.0 (5.4–11.9)	
**Frequency of listening to radio**			< 0.001*			< 0.001*			< 0.001*
Not at all	7971 (39.2)	64.5 (62.1–66.9)		6588 (49.4)	70.6 (67.9–73.1)		1383 (20.4)	37.6 (32.3–43.3)	
< once a week	4780 (24.9)	44.4 (41.2–47.6)		2988 (23.0)	59.5 (55.8–63.0)		1782 (28.2)	21.7 (18.0–26.0)	
≥ once a week	6837 (35.9)	31.1 (29.1–33.2)		3225 (27.6)	45.5 (42.0–49.0)		3312 (51.4)	16.8 (14.8–19.1)	
**Frequency of watching television**			< 0.001*			< 0.001*			< 0.001*
Not at all	10482 (53.7)	68.1 (65.8–70.3)		8971 (70.7)	71.9 (69.4–74.3)		1511 (22.3)	45.9 (40.7–51.2)	
< once a week	3367 (17.0)	31.1 (27.9–34.4)		1807 (12.6)	42.9 (38.5–47.3)		1560 (25.2)	20.2 (16.6–24.3)	
≥ once a week	5724 (29.3)	19.3 (17.4–21.2)		2316 (16.7)	28.9 (25.5–32.5)		3408 (52.5)	13.6 (11.5–16.0)	
***Enabling factors***									
**Access to health insurance**			< 0.001*			< 0.001*			< 0.001*
No	19235 (98.5)	48.1 (46.0–50.1)		12997 (99.4)	61.3 (58.7–63.8)		6238 (96.8)	22.9 (20.4–25.6)	
Yes	342 (1.5)	8.9 (5.5–13.9)		101 (0.6)	21.2 (12.2–34.4)		241 (3.2)	4.5 (2.1–9.5)	
**Distance to health facility**			< 0.001*			< 0.001*			< 0.001*
Big problem	6290 (32.0)	67.6 (64.7–70.3)		5272 (40.9)	74.8 (71.9–77.4)		1018 (15.6)	32.7 (26.9–39.0)	
Not a big problem	13284 (68.0)	38.0 (36.1–40.0)		7825 (59.1)	51.6 (49.0–54.2)		5459 (84.4)	20.3 (18.0–22.9)	
**Permission to visit health facility**			< 0.001*			< 0.001*			< 0.001*
Big problem	2339 (12.5)	74.5 (71.3–77.5)		1903 (15.3)	81.8 (78.7–84.5)		436 (7.2)	46.1 (38.8–53.6)	
Not a big problem	17227 (87.5)	43.6 (41.6–45.6)		11187 (84.7)	57.4 (54.8–59.9)		6040 (92.8)	20.4 (18.1–22.8)	
**Getting money for health services**			< 0.001*			< 0.001*			< 0.001*
Big problem	8969 (44.2)	57.0 (54.5–59.4)		6835 (50.9)	67.0 (64.3–69.5)		2134 (31.8)	27.3 (24.0–31.0)	
Not a big problem	10590 (55.8)	40.0 (37.8–42.2)		6254 (49.1)	55.0 (52.0–57.9)		4336 (68.2)	19.9 (17.3–22.8)	
**Companionship to health facility**			< 0.001*			< 0.001*			< 0.001*
Big problem	2803 (14.5)	72.9 (69.6–75.9)		2348 (18.6)	79.7 (76.5–82.6)		455 (6.9)	38.8 (30.7–47.6)	
Not a big problem	16763 (85.5)	43.2 (41.2–45.1)		10743 (81.4)	56.8 (54.3–59.3)		6020 (93.1)	21.0 (18.7–23.6)	
***Need factor***									
**Desire for pregnancy**			< 0.001*			< 0.001*			< 0.001*
Then	17502 (90.3)	49.1 (46.9–51.2)		11859 (91.8)	62.4 (59.8–64.9)		5643 (87.4)	23.2 (20.5–26.2)	
Later	1598 (7.6)	32.3 (29.0–35.8)		970 (6.6)	45.2 (40.0–50.6)		628 (9.4)	15.7 (12.7–19.2)	
No more	463 (2.1)	28.7 (24.3–33.5)		256 (1.6)	43.9 (37.2–50.9)		207 (3.2)	14.6 (10.4–20.1)	

* Significant at < 1% level

** Significant at < 5% level

n = Sample size (unweighted).

^a^ Weighted percentages for the multistage sampling probability

### Prevalence of ANC underutilization

**Fig 2:**
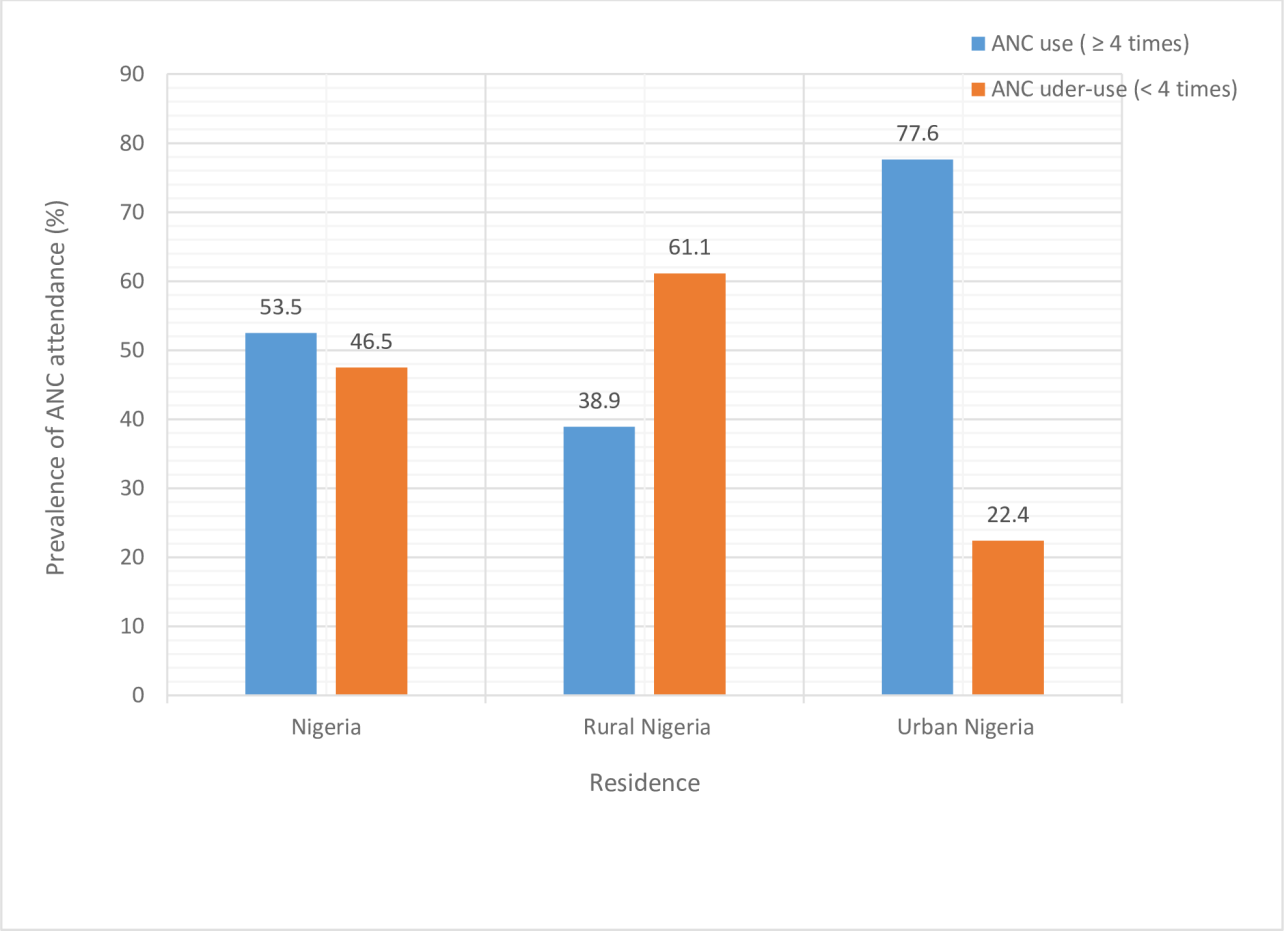
Prevalence of ANC underuse (< 4 times) and use (≥ 4 times) in Nigeria.

In the overall Nigerian population, a total of 10507 (53.5%) mothers reported having at least four ANC visits, 376 (1.8%) one-time visit, 744 (3.8%) two visits, 1363 (6.8%) three visits and 6662 (33.9%) had no antenatal care visit at all (data not shown on Tables). Furthermore, 9145 (46.5%) had attended ANC for less than the recommended minimum of four times–underuse. Only 38.9% of rural women had four or more antenatal visits as compared to 77.6% of urban women (Fig 2).

**Fig 3:**
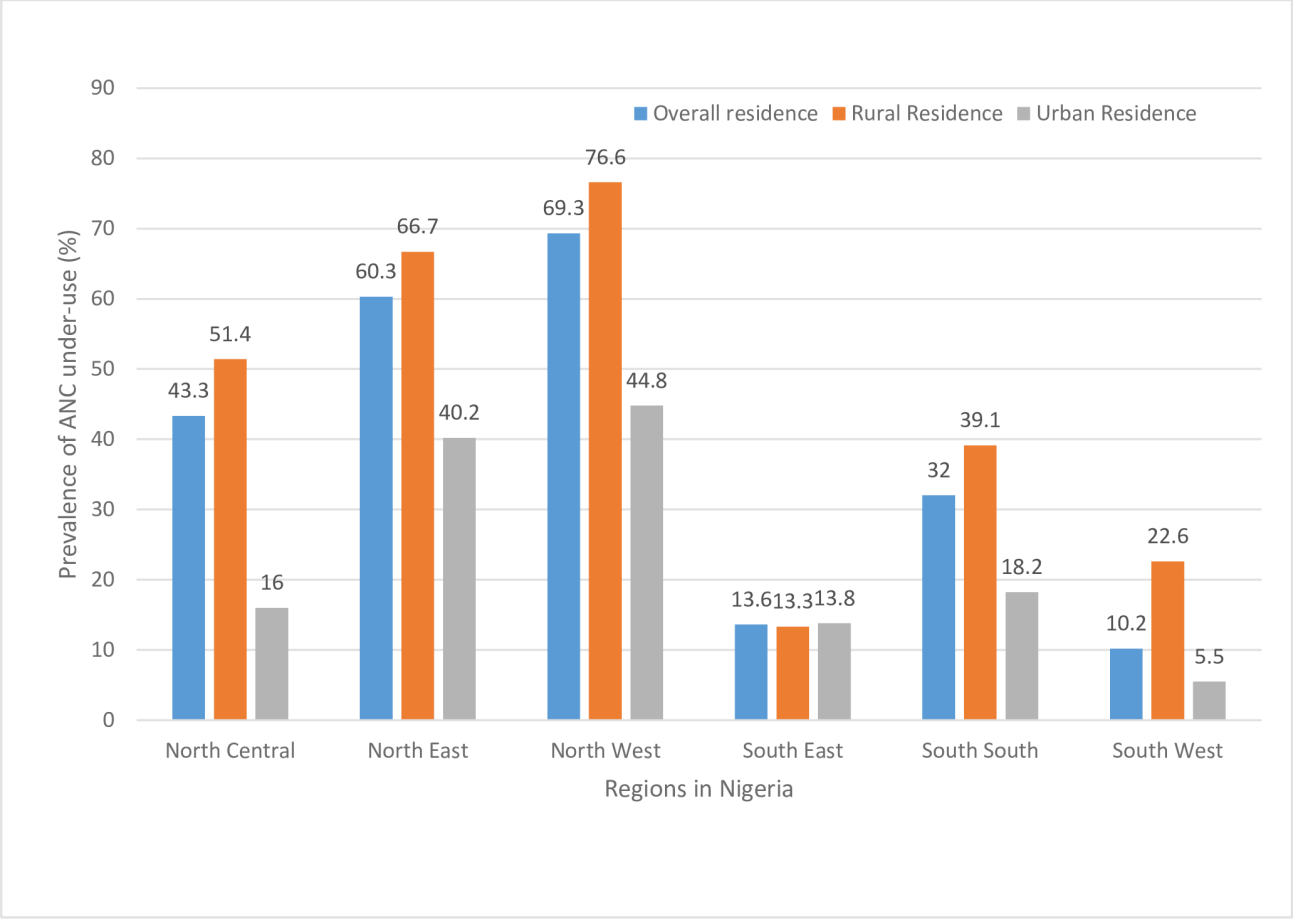
Regional differences in the prevalence of ANC underuse (< 4 times attendance) by residences in Nigeria.

Table 1 shows the prevalence of underuse of ANC among Nigerian women and a disaggregated status by rural and urban residences. The results showed that greater proportion of rural women (61.1%; 95% CI: 58.5–63.5) underutilized ANC compared to their urban counterpart (22.4; 95% CI: 20.0–25.1). Such difference also persisted when data was disaggregated by rural-urban residence for the different regions of the country except for South-East region where the prevalence of underutilization of ANC remains relatively similar (Fig 3). Furthermore, several socioeconomic variables were significantly associated with underuse of ANC as presented in Table 1.

### Factors associated with ANC underutilization

The result of Chi-Square test for the unadjusted association between ANC underuse and the various explanatory factors were equally presented in Table 1. Several factors were significantly associated with underuse of ANC in all the residences–overall, rural and urban (unadjusted association only). These factors included regions of residence, maternal education level, maternal working status, husband’s education level, wealth index, maternal age, maternal religion, birth order, presence of co-wives, final say on own healthcare, frequency of reading newspaper, frequency of listening to the radio, frequency of watching television, health insurance coverage, distance to health facility, permission to visit health facility, getting money to pay at health facility, companionship to attend facility and the desire for pregnancy.

Table 2 shows the results of the multivariable analyses for the overall, rural and urban residences in Nigeria. For the overall population, region of residence, types of residence, maternal education level, maternal working status, husband’s education level, wealth index, maternal age, birth interval, frequency of reading newspaper, frequency of listening to radio, frequency of watching television, and health insurance coverage remained significantly associated with underuse of ANC. Furthermore, distance to health facility, permission to attend facility and companion to get to the facility were significantly associated with ANC underuse.

**Table 2:** Factors associated with ANC underutilization (ANC attendance < 4 times).

Factors	Nigeria (overall)			Rural Nigeria			Urban Nigeria		
	AOR	95%CI	P-Value	AOR	95%CI	P-Value	AOR	95%CI	P-Value
**Region of residence**			<0.001*			< 0.001*			<0.001*
North-Central	3.50	2.35–5.16	<0.001*	3.44	1.92–6.21	< 0.001*	2.12	1.40–3.19	< 0.001*
North-East	3.06	2.03–4.60	<0.001*	2.41	1.31–4.43	0.005*	4.19	2.81–6.30	< 0.001*
North-West	4.92	3.29–7.35	<0.001*	4.25	2.31–7.77	< 0.001*	5.57	3.65–8.50	< 0.001*
South-East	1.17	0.78–1.76	0.457	0.88	0.48–1.63	0.678	1.89	1.19–3.00	0.007*
South-South	4.18	2.86–6.06	<0.001*	4.2	2.40–7.36	< 0.001*	4.72	3.01–7.40	< 0.001*
South-West	1.00	(Reference)	-	1.00	(Reference)	-	1.00	(Reference)	-
**Rural-urban residence**			0.010*						
Rural	1.27	1.06–1. 51	0.010*						
Urban	1.00	(Reference)	-						
**Maternal education level**			<0.000*			< 0.001*			0.028*
None	1.73	1.43–2.10	<0.000*	1.93	1.52–2.47	< 0.001*	1.44	1.10–1.87	0.008*
Primary	1.27	1.05–1.52	0.009*	1.35	1.09–1.67	0.007*	1.17	0.90–1.56	0.229
Secondary/higher	1.00	(Reference)	-	1.00	(Reference)	-	1.00	(Reference)	-
**Maternal working status**			<0.000*			< 0.001*			
Not working	1.25	1.12–1. 40	<0.000*	1.27	1.11–1.45	< 0.001*			
Working	1.00	(Reference)	-	1.00	(Reference)	-			
**Husband/partner’s education level**			<0.000*			< 0.001*			< 0.001*
None	2.14	1.84–2.49	<0.000*	2.03	1.72–2.43	< 0.001*	2.16	1.68–2.75	< 0.001*
Primary	1.11	0.95–1.28	0.156	1.05	0.88–1.22	0.563	1.15	0.88–1.49	0.282
Secondary/higher	1.00	(Reference)	-	1.00	(Reference)	-	1.00	(Reference)	-
**Wealth index**			<0.001*			< 0.001*			< 0.001*
Poor	2.17	1.76–2.64	<0.001*	2.17	1.68–2.81	< 0.001*	2.05	1.51–2.79	< 0.001*
Middle	1.30	1.09–1.55	0.002*	1.20	0.96–1.50	0.114	1.38	1.08–1.78	0.008*
Rich	1.00	(Reference)	-	1.00	(Reference)	-	1.00	(Reference)	-
**Maternal age**			0.005*			0.029*			0.012*
< 20 years	1.19	0.83–1.74	0.332	1.10	0.75–1.61	0.578	1.75	1.13–2.70	0.012*
20–34 years	1.17	1.07–1.29	0.001*	1.16	1.04–1.31	0.010*	1.25	1.03–1.49	0.015*
≥ 35	1.00	(Reference)	-	1.00	(Reference)	-	1.00	(Reference)	-
**Maternal religion**									0.006*
Islam							1.64	1.21–2.26	0.002*
Traditional/other							1.40	0.80–2.43	0.243
Christianity							1.00	(Reference)	-
**Preceding birth interval**			0.010*			0.002*			
< 24 months	1.17	1.04–1.30	0.010*	1.26	1.09–1.46	0.002*			
≥ 24 months	1.00	Reference	-	1.00	(Reference)	-			
**Birth type**						0.049*			
Single				1.46	1.00–2.15	0.049*			
Multiple				1.00	(Reference)	-			
**Frequency of reading newspaper/magazine**			<0.001*						0.002*
Not all	1.63	1.25–2.16	<0.001*				1.74	1.17–2.59	0.005*
< once a week	1.19	0.84–1.64	0.31				1.18	0.74–1.82	0.474
≥ once a week	1.00	(Reference)	-				1.00	(Reference)	-
**Frequency of listening to radio**			0.017*			0.026*			
Not all	1.24	1.05–1.44	0.010*	1.28	1.05–1.54	0.015*			
< once a week	1.21	1.04–1.41	0.016*	1.23	1.02–1.45	0.020*			
≥ once a week	1.00	(Reference)	-	1.00	(Reference)	-			
**Frequency of watching television**			<0.001*			< 0.001*			0.001*
Not all	1.46	1.22–1.74	<0.001*	1.50	1.20–1.88	0.001*	1.61	1.23–2.06	< 0.001*
< once a week	1.03	0.85–1.24	0.797	0.97	0.77–1.26	0.896	1.10	0.85–1.43	0.474
≥ once a week	1.00	(Reference)	-	1.00	(Reference)	-	1.00	(Reference)	-
**Access to health insurance**			0.009*						0.003*
No	2.32	1.24–4. 40	0.009*				3.41	1.53–7.58	0.003*
Yes	1.00	(Reference)	-				1.00	(Reference)	-
**Distance to health facility**			<0.001*			< 0.001*			0.001*
Big problem	1.66	1.45–1. 91	<0.001*	1.68	1.41–1.98	< 0.001*	1.42	1.14–1.79	0.001*
Not a big problem	1.00	(Reference)	-	1.00	(Reference)	-	1.00	(Reference)	-
**Permission to visit health facility**			<0.001*			< 0.001*			< 0.001*
Big problem	1.59	1.31–1.90	<0.001*	1.53	1.22–1.90	< 0.001*	1.80	1.30–2.49	< 0.001*
Not a big problem	1.00	(Reference)	-	1.00	(Reference)	-	1.00	(Reference)	-
**Getting money for health services**						0.040*			
Big problem				1.17	1.01–1.35	0.040*			
Not a big problem				1.00	(Reference)	-			
**Companion to health facility**			0.002*			0.003*			
Big problem	1.30	1.10–1.52	0.002*	1.32	1.09–1.56	0.003*			
Not a big problem	1.00	(Reference)	-	1.00	(Reference)	-			

* Significant at P < 0.05 level.

AOR: Adjusted Odds Ratio.–Vacant spaces in the Table indicate the factor in question did not attain statistical significance at p < 0.05 in the residence.

**Factors adjusted for but did not attain significance in Nigeria (overall):** Husband/partner’s working status, maternal religion, birth order, birth type, presence of co-wives, final say on own health, getting money for health services, desire for pregnancy.

**Factors adjusted for but did not attain significance in rural Nigeria**: Husband/partner’s working status, maternal religion, birth order, presence of co-wives, final say on own health, frequency of reading newspaper/magazine, access to health insurance, desire for pregnancy

**Factors adjusted for but did not attain significance in urban Nigeria**: Maternal working status, birth order, presence of co-wives, final say on own health, frequency of listening to radio, getting money for health services, desire for pregnancy

This study aimed at assessing the rural-urban differences in factors associated with ANC underuse in Nigeria and Table 2 similarly elaborates on these results. In rural Nigeria, a number of factors were associated with underuse of ANC. All the regions had greater odds of ANC underuse compared to the South-West region except the South-East which did not attain statistical significance. Women with no education (Adjusted odds ratio (AOR): 1.93; 95% CI: 1.11, 1.45) and those with primary education (AOR: 1.35; 1.09, 1.67) had greater odds of ANC underuse compared to their counterparts with a secondary/higher education. Similarly, women whose husband did not acquire any formal education (AOR: 2.03; 95% CI: 1.72, 2.43) and those who were not working (AOR: 1.27; 95% CI: 1.11, 1.45) had greater odds of ANC underuse (Table 2). The odds of ANC underuse were greater among mothers in poor households (AOR: 2.17; 95% CI: 1.68, 2.81), as well as mothers aged 20–34 years (AOR: 1.16, 95%CI: 1.04, 1.31). Preceding birth interval <24 months (AOR: 1.26; 95% CI: 1.09, 1.46) and singleton birth type (AOR: 1.46; 95%CI: 1.00, 2.15) were equally associated with increased odds of ANC underuse. Lack of access to radio and television, perceived big problems with regards to distance, permission, being accompanied and getting money to visit healthcare facility similarly increased the odds of ANC underuse.

In urban Nigeria, some of the factors associated with underuse of ANC were comparable to those found in rural residence (Table 2). The regional differences showed that all the regions had lower use of four or more antenatal visits compared to the South-West. Greater odds of ANC underuse were associated lack of maternal education (AOR: 1.44; 95% CI: 1.10, 1.87), living in poor households (AOR: 2.05; 95% CI: 1.51, 2.79) and lack of husband’s education (AOR: 2.16; 95% CI: 1.68, 2.75). Similarly, mothers in the age categories <20 years (AOR: 1.75; 95% CI: 1.13, 2.70) and 20–34 year (AOR: 1.25; 95% CI: 1.03, 1.49) had greater odds of ANC underuse compared to their counterparts in the ≥ 35-year category. Not having access to television, perceived big problems in relation to distance, and permission to visit healthcare facility remained significantly associated with ANC underuse (Table 2).

In contrast to the rural residence, maternal working status, birth interval, birth type, access to radio, the problem of money and being accompanied to a health facility were not significantly associated with underutilization of ANC in urban Nigeria. However, maternal religion was significantly associated with ANC underuse in urban areas only and women professing Islamic religion had 64% higher odds of underutilization than their Christian counterparts (AOR: 1.64; 95%CI: 1.21, 2.26). Unlike in rural residence, urban mothers who reported not reading newspaper/magazine at all (AOR: 1.74; 95%CI: 1.17, 2.59), those who did not enjoy access to health insurance coverage (AOR: 3.41; 95%CI: 1.53, 7.58) as well as mothers who resided in the South-East region (AOR: 1.89; 95%CI: 1.19, 3.00) had greater odds of ANC underuse.

## Discussion

This study assessed the prevalence and factors associated with ANC underuse in Nigeria with an emphasis on the rural-urban differences, using the most recent nationally representative data– 2013 NDHS. A previous study [20], based on the 2008 NDHS, has reported the determinants of “urban-rural differentials” of ANC utilization in Nigeria”. However, the determinants of ANC reported in the study were relevant only to the overall Nigerian population since the multivariable analysis conducted was limited to the overall data (not stratified by rural-urban residences) [20]. The factors found to be associated with ANC use in the study–maternal age, residence, region, maternal education level, partner education level, distance to health facility, employment status, and wealth index [20]–retained their statistical significance for the overall Nigerian population in the present study. However, given the comprehensive selection of our independent variables, we found several other factors, including frequency of media exposure (newspaper, television, and radio) and health insurance coverage, to be associated with ANC underuse in Nigeria (overall population). Moreover, we disaggregated the overall data by rural and urban residence and compared the results from the two residences.

Our findings indicate that rural-urban differences exist in the prevalence of ANC under-use in Nigeria. Generally, rural mothers had a greater prevalence of underuse compared to their urban counterparts. We equally observed regional differences with the South-East and South-West regions having the lowest prevalence in rural and urban residences, respectively. On the other hand, the North-West region had the highest prevalence of ANC underuse both in rural and urban residences. Following multivariable analyses, six factors–maternal working status, birth interval, birth type, the frequency of listening to radio, getting money to pay and companionship to healthcare facility–attained statistical significance in rural but not in urban residence. Conversely, three factors namely, maternal religion, the frequency of reading newspaper and health insurance coverage were significant in urban but not in rural residence. Eight other factors–region of residence, maternal and husband/partner’s education level, wealth index, maternal age, frequency of watching television, distance to health facility and permission to visit health facility–were consistently associated with ANC underuse in both rural and urban residences. Thus, varying degrees of rural-urban differences in factors associated with ANC underuse exist in Nigeria.

The findings that rural residence had significantly higher prevalence and greater odds of ANC underuse than urban Nigeria are consistent with the report of previous studies both in Nigeria and elsewhere [16, 20, 26]. Inequitable access to healthcare facilities/services in rural compared to urban areas in Nigeria may explain these differences. Due to low coverage, rural residents are generally disadvantaged in respect of access to healthcare facilities/services in Nigeria [4, 32, 33]. Where facilities exist, inaccessibility due to the poor road network, lack of efficient transport system and distance barrier may co-exist in the residence [23, 34]. These possibly explain why being accompanied to health facility assumed a significant status as a risk factor for ANC underuse in rural Nigeria. Interestingly, distance to health facility equally attained statistical significance in all residences but with greater odds in rural areas. Traditional beliefs, poorly equipped/staffed healthcare facilities, as well as poor socioeconomic circumstances are some of the other factors which may contribute to underutilization of maternal healthcare services like ANC in rural residence [23, 34].

Whether in rural or urban residence, all the regions in Nigeria, except the rural South-East, had greater odds of ANC underuse compared to the South-West (the reference category). These findings agree with our Chi-Square test which revealed that the South-East and the South-West regions had the lowest prevalence of ANC underuse in rural and urban residences, respectively. The present study, therefore, lends credence to previous evidence showing better ANC utilization in South-West and South-East regions in the country [4, 20, 35]. Differences in socioeconomic development, educational attainment and access to healthcare facilities in the various regions may partly explain the disparities observed in this study [4, 36]. For example, the South-West region relatively enjoys a greater access to education and healthcare facilities than other regions in the country. On the other hand, the northern regions (North-Central, North-East, and North-West) are educationally and socioeconomically disadvantaged [36]. Socioeconomic disadvantage occasioned by environmental degradation, infrastructure deficits, and low employment level has equally been reported in the South-South region of the country [37]. These may contribute to low maternal healthcare services utilization in the regions.

Furthermore, security challenges in parts of North-East, North-West (insurgency) and South-South (militancy) regions, especially, in the rural areas may have contributed to regional variations in this study [4, 37]. However, in urban residence, our study indicates that mothers professing Islamic religion had greater odds of ANC underuse compared to their Christian counterparts. A previous study has made a similar report in respect of maternal healthcare services utilization among Muslim mothers in Nigeria [6]. Given that Islam is more prominent in parts of northern regions in Nigeria [36, 38], it is likely that the finding of low ANC utilization among Muslim mothers contributed to the regional differences observed in this study, especially in urban residence. Religious obligations which require that Muslim women avoid undue exposure of their bodies have been suggested as the reason for the finding [38, 39].

Financial and socioeconomic-related factors were overwhelmingly associated with ANC underuse in this study. For instance, in both rural and urban residences, mothers classed in the poor wealth index category had at least two-fold increased odds of ANC underuse compared to their counterparts in rich households. These results agree with previous studies for the overall Nigerian population [19, 35] and highlight the negative impacts of poverty/low socioeconomic status on ANC utilization in Nigeria. This explanation may equally be relevant to the finding in rural residence which showed increased odds of ANC underuse in ‘not working mothers’ since lack of employment may translate to a low financial capability for healthcare services. The majority of Nigerian population still live below the poverty level, hence, bridging socioeconomic disparities in access to healthcare services should be a priority in the country [4].

For example, provision of free maternal healthcare services may improve ANC utilization across socioeconomic divides in the country, particularly in rural Nigeria where the problem of getting money increased the odds of ANC underuse. Similarly, our findings suggest that universal health insurance coverage remains an important entry point to improving ANC attendance, especially in urban Nigeria where lack of access to health insurance increased the odds of ANC underuse by nearly 3.5-fold. However, the results of our descriptive statistics show that health insurance coverage was unacceptably low at 1.5%, 0.6% and 3.2% in the overall, rural and urban residences, respectively. Future interventions need to prioritize this finding for an improved use of ANC in Nigeria.

Furthermore, lack of maternal and husband education increased the odds of ANC underuse in Nigeria, the rural-urban residence notwithstanding. These findings compare well with those of studies (overall national population only) in Nigeria [16, 20], Indonesia [26] and Timor-Leste [27]. However, unlike husband’s education, the results for maternal education exhibited a dose-response relationship similar to the findings of previous studies in Nigeria and Timor-Leste [16, 27]. This result suggests that low maternal education was better than none in the utilization of ANC services in Nigeria and may be an indication of the relative importance of maternal education over husband’s education. Besides its contribution to improved socioeconomic status and financial capability, education may empower women to make informed and responsible decision in matters of their wellbeing and this may contribute to increased utilization of healthcare services including ANC attendance [39, 40]. Similarly, better-educated husbands would more readily understand the importance of ANC and, thus support their wives for increased utilization of the services [39]. Lack of- or low maternal, as well as husband's education probably explains why getting permission to visit healthcare facility remained a significant challenge in all residences in Nigeria.

The contribution of media exposure to the odds of ANC underuse was equally highlighted in this study. Based on our findings, not watching television at all contributed to increased odds of ANC underuse in all residences in Nigeria. On the other, none or low frequency of listening to radio was significantly associated with higher odds of ANC underuse in rural residence while none or low frequency or reading newspapers was a risk factor in urban residence. These findings are similar to those of a study in Nepal [29], and they underscore the important role of media exposure in healthcare services utilization. Second, the results indicate that some media may be more effective than others in reaching different population groups in Nigeria for ANC utilization. This observation may find relevance in designing appropriate behavior change communication models [41] for improved uptake of not only ANC but other healthcare services in Nigeria. For, instance, health information through the newspaper would not be effective in rural residence as found in this study. Thus, it may be appropriate, for instance, to encourage rural women to frequently listen to radio for a better exposure to healthcare information.

### Strengths and limitations

The use of recent nationally representative datasets, high response rate and rural-urban disaggregation of data are some of the strengths of this study. Hence, findings are current and generalizable. Also, given the large sample size for the survey, data disaggregation does not undermine generalizability. Other notable strengths include low missing data and application of complex samples statistics in data analysis to adjust for the sample weight and cluster design of the dataset used. The use of a well-regarded behavioral model as a conceptual framework means relevant independent variables were comprehensively assessed in this study. To the best of our knowledge, this is the first nationally representative study to comprehensively assess rural-urban differences in the prevalence and factors associated with ANC underuse in Nigeria. Nevertheless, a few limitations need to be considered when the results of this study are being interpreted. First, a causal relationship could not be estimated owing to the cross-sectional design of the survey. Second, the dataset analyzed in this study were self-reported, collected retrospectively and so prone to social desirability and recall biases.

## Conclusions

This study assessed the prevalence and factors associated with ANC underuse in Nigeria with a focus on the rural-urban differences. Findings indicate that the prevalence of ANC underuse differs between rural and urban residences in Nigeria, and rural residence had significantly higher prevalence. Regional differences were equally observed in both residences with the South-East and South-West regions having the lowest prevalence in rural and urban residences, respectively. On the other hand, the North-West region had the highest prevalence of ANC underuse, rural-urban residence notwithstanding. Factors associated with ANC underuse differ to varying degrees in both residences. The need to address the rural-urban as well as regional disparities in ANC use was highlighted in this study. Generally, rural residence, northern regions as well as the South-South region of the country require a greater priority for increased ANC utilization. Specifically, however, the need for targeted interventions for women professing Islamic religion (especially in urban residence), uneducated mothers and fathers (in all residences) as well as poor mothers (in all residences) was revealed.

Practical and implementable interventions such as equitable access to healthcare facilities need to be pursued in rural Nigeria. Healthcare/ANC facilities would need to be sited within manageable distance for ease of accessibility. Also, availability of a universal health insurance coverage (particularly, in urban residence), as well as the provision of free ANC services (especially in rural residence) may contribute in addressing financial constraints associated with ANC attendance in Nigeria. Intervention efforts focused on improved access to-/knowledge of family planning services in rural residence may prove beneficial in addressing the challenge of a short interval between births, and subsequently, ANC underuse. Also, appropriate selection of media services for health promotion purposes based on findings in this study should form part of a holistic approach to addressing the challenge of ANC underuse in Nigeria. Lastly, as part of a long-term strategy, education of at least a secondary school level needs to be promoted in all residences in Nigeria.
